# Supplementary material for: Studying the Bulk and Contour Ice Nucleation of Water Droplets via Quartz Crystal Microbalances
Source: Micromachines (Basel). 2021 Apr 20;12(4):463. doi: 10.3390/mi12040463 (PMC8074365; doi:10.3390/mi12040463)
Supplement: Supplementary file 1 [file micromachines-12-00463-s001.zip › Supporting information_freezing mode.pdf]

Studying the bulk and contour ice nucleation of water droplets via quartz crystal microbalances

Supporting information

Karekin D. Esmeryan[1,*] and Nikolay I. Stoimenov[2]

[1] Acoustoelectronics Laboratory, Georgi Nadjakov Institute of Solid State Physics, Bulgarian Academy of Sciences, 72, Tzarigradsko Chaussee Blvd., 1784 Sofia, Bulgaria
[2] Department of Distributed Information and Control Systems, Institute of Information and Communication Technologies, Bulgarian Academy of Sciences, Acad. G. Bonchev Street, Bl.2, Sofia, Bulgaria
*Correspondence: karekin_esmerian@abv.bg; Tel.: +359-2-979 5811 (K.E.)

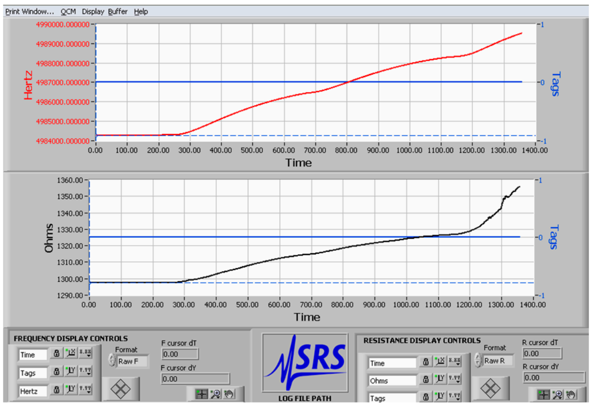

a)

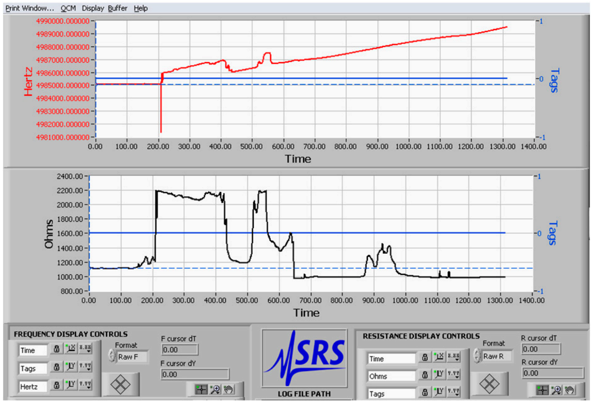

b)

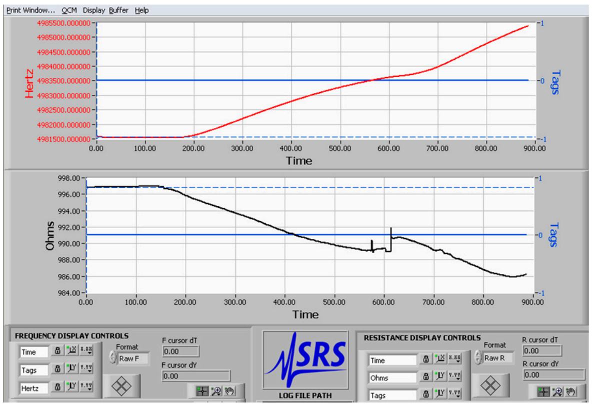

c)

Figure S1. Reproducibility of the sensor response induced by thermal fluctuations (cooling of the system) during the bulk mode nucleation assays. The observed fluctuations in Figure S1b are attributed to temperature-induced mechanical stresses in the soot coating.

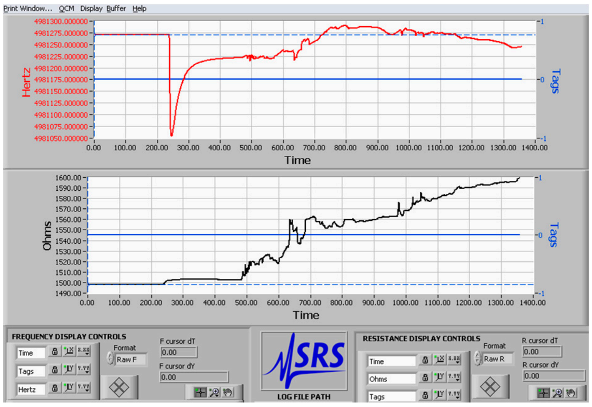

a)

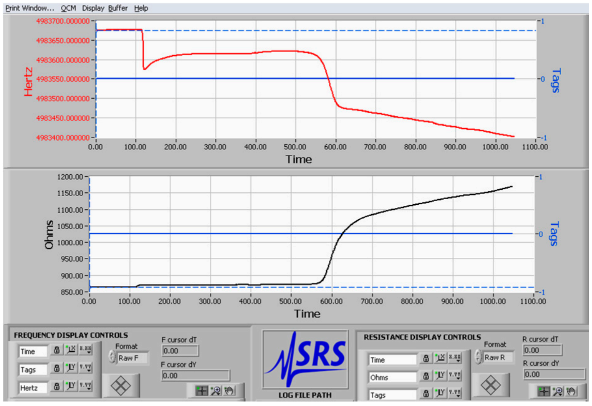

b)

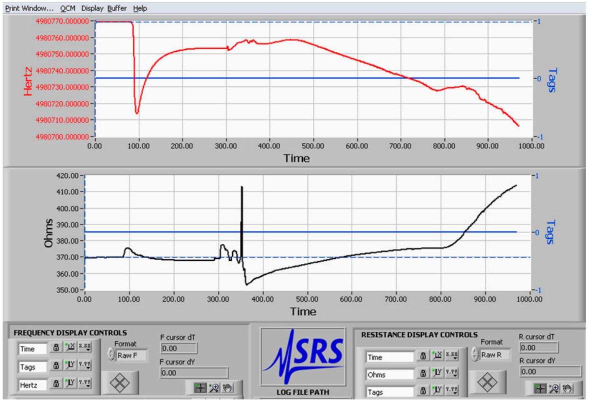

c)

Figure S2. Reproducibility of the sensor response induced by thermal fluctuations (cooling of the system) during the contour mode nucleation assays. The observed fluctuations in both resonance frequency and series resistance are attributed to the mismatch between the elastic properties of water and the soot coating.

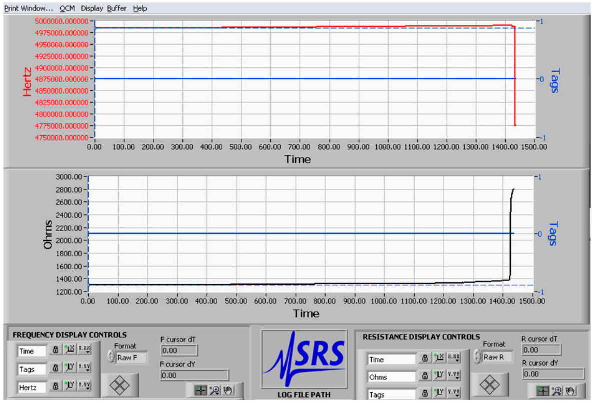

a)

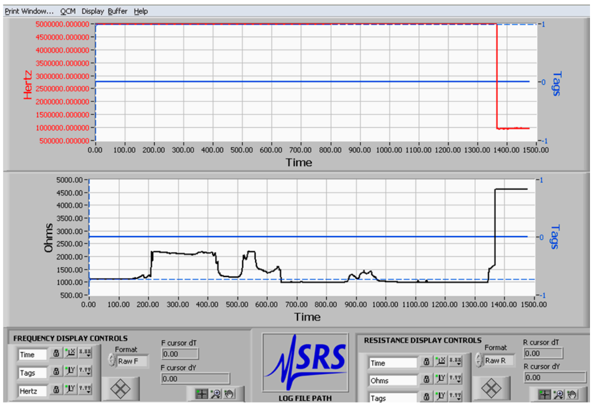

b)

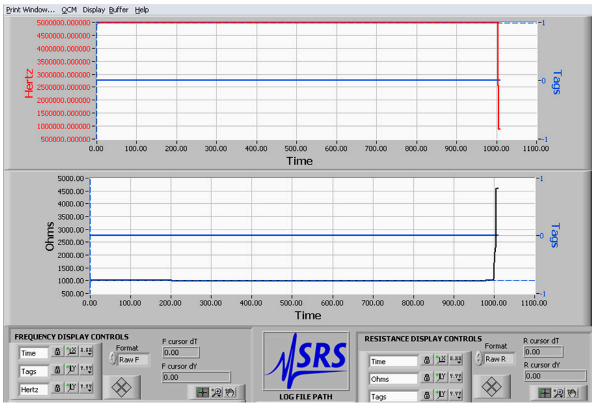

c)

Figure S3. Reproducibility of the sensor response induced by the recalescent freezing of the water droplet during the bulk mode nucleation assays. Once the solid-liquid interface freezes (within 1-2 s), the QCM gets out-of-resonance.

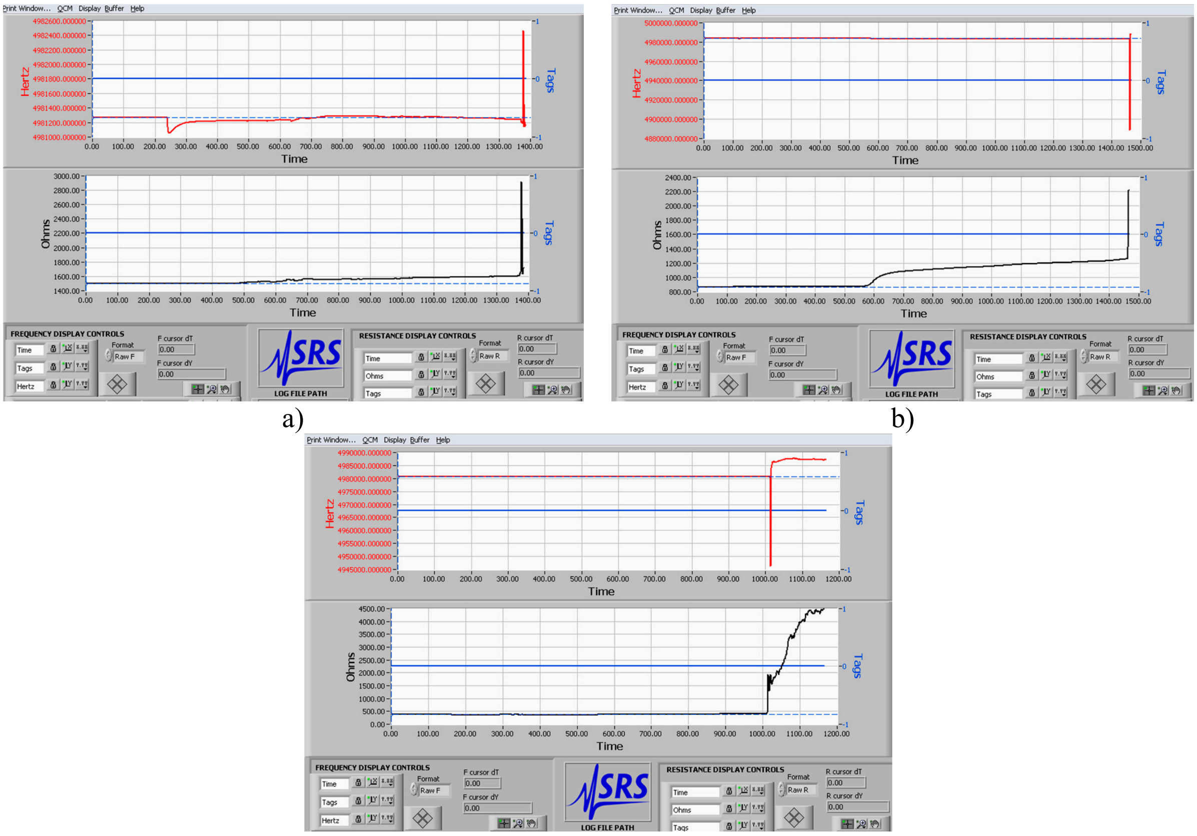

c)

Figure S4. Reproducibility of the sensor response induced by the recalescent freezing of the water droplet during the contour mode nucleation assays. Once the solid-liquid interface and the droplet's entire outer shell freeze, the QCM is still capable of generating oscillations for a certain time range (62-149 s) prior to getting out-of-resonance (see the main article).
